# Stratified Prognostic Value of Pathological Response to Preoperative Treatment in yp II/III Rectal Cancer

**DOI:** 10.3389/fonc.2021.795137

**Published:** 2021-12-16

**Authors:** Yanpeng Yang, Hao Xu, Guowei Chen, Yisheng Pan

**Affiliations:** Department of Gastroenterology, Peking University First Hospital, Beijing, China

**Keywords:** rectal cancer, survival, neoadjuvant (chemo)radiotherapy, tumor regression grade (TRG), prognosis

## Abstract

**Aim:**

Accumulated studies have verified that tumor regression is associated with the prognosis of rectal cancer. However, stratified analysis within a certain stage is still unknown. The purpose of our study was to assess the impact of pathologic response on the survival of stageII and III rectal cancer patients after neoadjuvant chemoradiotherapy (nCRT).

**Methods:**

Clinicopathologic characteristics and tumor regression scores (TRS) were assessed in 236 rectal cancer patients who treated with nCRT followed by surgery. Survival analysis was performed using Cox proportional hazards models.

**Results:**

Among these patients, the stage of 88 patients was ypII, and 91 patients were with the stage of ypIII. The median follow-up time was 59.8 months. TRS was not an independent prognostic factor in ypII patients while it was significantly associated with the prognosis of ypIII patients (5-year survival rate 67.2% vs. 42.5%, *P* < 0.001). Furthermore, ypIII patients with the response to nCRT had similar survival to that of ypII patients (5-year survival rate 67.2% vs. 70.5%, *P* = 0.56). For ypIII patients, multivariable analysis showed that well differentiation, negative surgical margin, and the administration of adjuvant chemotherapy were associated with better survival. The surgical margin and differentiation were prognostic factors for ypII patients.

**Conclusions:**

ypIII rectal cancer patients with poor response to preoperative treatment are at high risk of worse oncological outcomes.

## Introduction

Neoadjuvant chemoradiotherapy (nCRT) was recommended in the 1990s to treat the patients with locally advanced rectal cancer. Compared to the conventional surgery combined with postoperative therapy, nCRT has been proved more effective in local control, downstaging, and sphincter sparing (1, 2). The pathologic stage of the resection specimen (yp) is the strongest prognostic factor for patients who underwent neoadjuvant therapy. The ypI patients usually possess long-term survival and local control, while 30% of ypII and III rectal cancer patients will suffer from relapse (3).

Therefore, physicians need to find a prognostic factor that can precisely predict the survival of high-risk individuals. It will be benefit to decide the adjuvant regimen and appropriate surveillance intervals. Until now, the most accurate method for evaluating the response is the histological change of the resected specimen. These changes include cytologic and stromal alterations, such as cytoplasmic vacuolation and mucus lakes at the site of previous tumors (4, 5). Tumor regression can range from no evidence of therapeutic effect to complete response with no residual cancer cells. To conveniently describe the response to nCRT, the tumor regression score (TRS) was introduced to evaluate the degree of remission. According to previous researches, patients with pathologic complete remission (pCR) generally have superior long-term survival and at low risk of relapse. Patients with moderate, minimal, or no response have progressively worse outcomes (4, 6).

Accumulated trials have verified that well pathological responses, such as pCR and ypI, are associated with better outcomes. However, the survival of ypII and III rectal cancer patients are variable. They maybe experience recurrence at a rate of 20–30% (7–11). Thus, only depending on yp stage is not enough to identify the high-risk population. Many published studies have confirmed the prognostic value of the response to neoadjuvant treatment (4), but a stratified analysis within a certain stage is still unknown. We hypothesized that well response to nCRT would be associated with improved survival among specific patients. Therefore, the purpose of our study was to assess the impact of nCRT response on the survival of ypII and ypIII rectal cancer patients, which could help to identify high-risk rectal cancers.

## Methods

### Patients and Data Sources

A total of 256 pathologically confirmed rectal cancer patients who were treated with nCRT followed by surgery in Peking University First Hospital between 2008 and 2019 were collected in our study. The medical records and surveillance data were obtained prospectively. This had been approved by the institutional review board. Exclusion criteria included incomplete nCRT or surgery, stage IV disease, history of other cancers, and insufficient data.

### Treatment

Rectal cancer with clinical stage of T3-4 or N+ was defined as locally advanced rectal cancer. Treatments for locally advanced rectal cancer patients were decided by multidisciplinary team (MDT) discussions. The team consisted of professional oncologists, surgeons, radiologists, and pathologists. The patients involved in this research received concurrent chemotherapy with radiation, usually oral capecitabine or intravenous 5-FU and a long course of 50.4 Gy radiation in 25 fractions, followed by surgery with curative intent. Surgeries complied with the principle of total mesorectal excision (TME). And the interval between the last treatment and surgery was about 8 weeks for all the patients. All patients were encouraged to receive adjuvant chemotherapy after surgery. The regimens of adjuvant chemotherapy were CapeOX or FOLFOX. And up to 6 months of perioperative chemotherapy was recommended.

Response to nCRT was evaluated by experienced pathologists without knowing the outcomes of the patients. The system used to grade the tumor response was recommended by the AJCC Cancer Staging Manual (8th Edition) and the College of American Pathologists (CAP) guidelines: tumor regression score 0 (TRS 0) (complete response), no remaining viable cancer cells; TRS 1 (moderate response), only small clusters or single cancer cells remaining; TRS 2 (minimal response), residual cancer remaining, but with predominant fibrosis; TRS 3 (poor response), minimal or no tumor kill, extensive residual cancer.

### Statistical Analysis

Clinicopathologic characteristics and oncologic outcomes of the populations were collected and analyzed. The association between these factors and TRS was assessed by the chi-square, Fisher exact, and Mann-Whitney *U* tests, as appropriate. Survival was estimated using the log-rank test. Variables were selected into the multivariable model depend on statistical significance (*P* < 0.2), and the stepwise Cox regression model was used. All analyses were carried out with IBM SPSS version 27.0. Statistically significant was considered when a two-tailed *P*-value less than 0.05.

## Results

### Patient Characteristics

A total of 256 rectal cancer patients were involved in our research, and the prognosis and characteristics were further analyzed. The median follow-up time was 59.8 months, and the 3- and 5-year overall survivals of the entire population were 78.5% and 69.3%, respectively. After nCRT, 31 patients (12.1%) achieved pCR (TRS 0), whereas 58(22.7%), 96 (37.5%), and 71 (27.7%) patients had the TRS of 1, 2, and 3, respectively. The pathological differentiation in most patients was moderate and poor (69.8%). After surgery, 40.7% of patients received adjuvant chemotherapy.

### Characteristics and Survival Analysis of ypII

Stratified by tumor regression scores, clinical and pathological characteristics of ypII rectal cancer patients were summarized in **Table 1**. Eighty-eight patients were staged ypII, and most of the patients were at ypT3 stage (78.4%). In addition to this, only 37.5% of ypII patients received adjuvant chemotherapy. And predictors of pathologic response were gender (*P* = 0.03) and tumor size (*P* = 0.03).

**Table 1:** Clinicopathologic characteristics of ypII patients.

Variables	Tumor regression score			*P* value
	1	2	3	
**Age (88)**				0.19
≥65 (34)	13	11	10	
<65 (54)	14	28	12	
**Gender (88)**				0.03
Male (59)	23	21	15	
Female (29)	4	18	7	
**BMI, kg/m^2^ (88)**				0.38
< 24 (53)	15	22	16	
≥24 (35)	12	17	6	
**Clinical T stage (88)**				0.98
T1 (6)	1	3	2	
T2 (15)	5	6	4	
T3 (38)	13	16	9	
T4 (29)	8	14	7	
**Clinical N stage (88)**				0.20
N0 (22)	4	12	6	
N1 (56)	17	24	15	
N2 (10)	6	3	1	
**Clinical stage (88)**				0.33
II (22)	4	12	6	
III (66)	23	27	16	
**Procedure (88)**				0.45
LAR (56)	20	23	13	
APR (21)	3	12	6	
Combined resection (11)	4	4	3	
**Distance from anal verge, cm (87)**				0.06
≥5 (62)	24	25	14	
< 5 (25)	3	14	8	
**Tumor size, cm**				0.03
≥3.5 (27)	3	16	8	
< 3.5 (61)	24	23	14	
**Pathological T stage (88)**				0.69
T3 (69)	21	32	16	
T4 (19)	6	7	6	
**Histological differentiation (83)**				0.90
Well (4)	1	3	0	
Moderate (64)	18	29	17	
Poor (15)	5	6	4	
**Positive lymphovascular invasion**	1	3	2	0.77
**Positive surgical margin**	1	4	4	0.28

**Figure 1:**
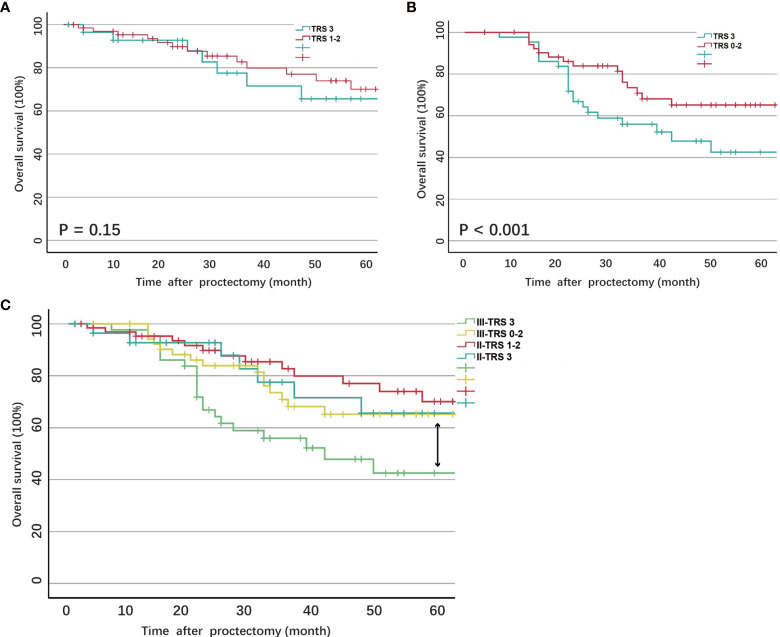
**(A)** Overall survival of ypII patients stratified by tumor regression score; **(B)** Overall survival of ypII patients stratified by tumor regression score; **(C)** Overall survival of ypIII patients compared with ypII patients stratified by tumor regression score.

The 5-year survival rate of ypII patients was 70.5%, with a median survival of 101.4 months. Variables associated with survival after surgery in ypII patients were illustrated in **Table 2**. TRS 1 and 2 were grouped and compared with TRS 3. In the univariable analysis, we found that the TRS was associated with survival (*P* = 0.03). However, there was no statistical difference between them with multivariate analysis (**Figure 1A**, *P* = 0.15). In univariable and multivariable analysis, only the histologic differentiation (HR 4.17, 95% CI 0.71-6.25, *P* = 0.024) and surgical margin (HR 2.78, 95% CI 0.35–5.26, *P* < 0.001) remained difference significantly.

**Table 2:** Analysis of factors associated with overall survival for ypII patients.

Variables	Median survival (m)	5-year Survival (%)	Log-rank test	Cox multivariate test		
				HR	95% CI	*P*
**Age**			0.59			
≥65	80.2	69.0				
<65	101.4	71.2				
**Gender**			0.72			
Male	92.1	79.6				
Female	85.6	75.4				
**BMI, kg/m^2^**			0.61			
< 24	97.4	73.0				
≥24	74.5	68.6				
**Clinical stage**			0.06			
II	98.7	82.0				
III	83.6	70.5				
**Procedure**			0.04			
LAR	103.5	81.6		Ref		
APR	90.3	65.4		1.20	0.82-3.67	0.21
Combined resection	77.5	43.8		1.32	0.45-1.79	0.056
**Distance from anal verge**			0.91			
≥5 cm	101.3	72.7				
< 5 cm	87.5	69.1				
**Tumor size, cm**			0.68			
≥3.5	84.4	69.0				
< 3.5	104.1	71.7				
**Pathological T stage**			0.34			
T3	101.0	70.5				
T4	82.8	70.0				
**Histological differentiation**			0.006			
Well/moderate	111.7	82.7		Ref		
Poor	71	70.5		4.17	0.71-6.25	0.024
**Lymphovascular invasion**			0.64			
Negative	100.7	83.3				
Positive	49.2	69.5				
**Surgical Margin**			<0.001			
Negative	98.3	76.5		Ref		
Positive	NR	25.0		2.78	0.35-5.26	<0.001
**Adjuvant chemotherapy**			0.72			
No	102.7	69.6				
Yes	66.6	71.9				
**Tumor regression score**			0.03			
0-2	103.8	72.3		Ref		
3	80.6	65.2		1.16	0.83-2.77	0.15

NR, not reached.

### Characteristics and Survival Analysis of ypIII

Stratified by tumor regression scores, clinical and pathological characteristics of ypIII rectal cancer were summarized in **Table 3**. Ninety-one patients were staged ypIII. Four patients achieved a local pathologically complete response, whereas the lymph nodes harvested were positive. The predictors of pathologic response were the clinical nodal status (*P* = 0.03), stage (*P* = 0.047), and the pathologic T stage (*P* < 0.001).

**Table 3:** Clinicopathologic characteristics of ypIII patients.

Variables	Tumor regression score				*P* value
	0	1	2	3	
**Age (91)**					0.90
≥65 (34)	1	9c	17	7	
<65 (54)	3	15	25	14	
**Gender (91)**					0.40
Male (55)	2	18	23	12	
Female (36)	2	6	19	9	
**BMI, kg/m^2^ (91)**					0.94
< 24 (59)	3	16	26	14	
≥24 (32)	1	8	16	7	
**Clinical T stage (91)**					0.38
T1	0	0	2	1	
T2	2	4	5	6	
T3	2	11	26	10	
T4	0	9	9	4	
**Clinical N stage (89)**					0.03
N0	3	3	8	5	
N1	1	11	26	14	
N2	0	9	7	2	
**Clinical stage (89)**					0.047
II (19)	3	3	8	5	
III (70)	1	20	33	16	
**Procedure (91)**					0.90
LAR (60)	3	15	29	13	
APR (27)	1	8	12	6	
Combined resection (4)	0	1	1	2	
**Distance from anal verge, cm (91)**					0.91
≥5 (61)	3	17	28	13	
< 5 (30)	1	7	14	8	
**Tumor size, cm (91)**					0.71
≥3.5 (27)	1	6	15	5	
< 3.5 (64)	3	18	27	16	
**Pathological T stage (91)**					<0.001
T0(4)	4	0	0	0	
T1(2)	0	1	1	0	
T2(19)	0	6	10	3	
T3 (60)	0	14	29	17	
T4 (6)	0	3	2	1	
**Pathological N stage**					0.94
N1 (59)	3	16	26	14	
N2 (32)	1	8	16	7	
**Histological differentiation (89)**					0.18
Well (6)	1	2	3	0	
Moderate (56)	2	16	27	11	
Poor (27)	0	6	11	10	
**Positive Lymphovascular invasion**	0	1	4	3	0.63
**Positive surgical margin**	0	0	2	3	0.20

The 5-year survival rate of ypIII patients was 63.6%, with a median survival of 79.8 months. Variables associated with survival after surgery in ypIII patients were illustrated in **Table 4**. In the same way, TRS 0, 1, and 2 were grouped and compared with TRS 3 for analysis. TRS was associated with survival in the univariable analysis (*P* < 0.001). There was also a significant difference between them in multivariate analysis (HR 2.63, 95% CI 1.12-5.88, *P* < 0.001, **Figure 1B**). Besides, younger age, well histological differentiation, low anterior resection, negative surgical margin, and the completion of adjuvant chemotherapy were associated with better survival in univariable analysis. Negative surgical margin, well differentiation, and the presence of adjuvant chemotherapy remained statistically significant in multivariable analysis.

**Table 4:** Analysis of factors associated with overall survival for ypIII patients.

Variables	Median Survival (m)	5-year survival (%)	Log-rank test	Cox multivariate test		
				HR	95% CI	*P*
**Age**			0.04			
≥65	69.3	64.0		Ref		
<65	75.7	70.1		0.96	0.23-2.89	0.56
**Gender**			0.93			
Male	79.3	62.3				
Female	61.0	64.7				
**Clinical stage**			0.12			
II	78.8	76.3				
III	69.3	62.5				
**Procedure**			0.013			
LAR	82.1	75.6		Ref		
APR	64.5	63.2		1.05	0.52-1.33	0.06
Combined resection	NA	–				
**Distance from anal verge**			0.80			
≥5 cm	79.0	61.7				
< 5 cm	58.2	65.0				
**Tumor size, cm**			0.51			
≥3.5	73.5	65.4				
< 3.5	68.3	61.2				
**Pathological T stage**			0.32			
T0	NA					
T1	NA					
T2	78.5	62.5				
T3	66.3	52.7				
T4	NR					
**Pathological N stage**			0.06			
N1	79.4	81.8		Ref		
N2	64.8	36.8		1.26	0.33-2.89	0.14
**Histological differentiation**			0.003			
Well/moderate	68.2	71.4		Ref		
Poor	53.4	60.6		1.79	1.09-2.94	0.02
**Lymphovascular invasion**			0.64			
Negative	81.2	80.1				
Positive	42.8	56.3				
**Surgical Margin**						
Negative	79.6	78.3	<0.001	Ref		
Positive	NA	0		3.57	0.56-6.25	0.016
**Adjuvant chemotherapy**			<0.001			
No	63.7	43.5		Ref		
Yes	82.6	85.4		0.35	0.13-0.95	0.007
**Tumor regression score**			<0.001			
0-2	78.6	67.2		Ref		
3	56.0	42.5		2.63	1.12-5.88	<0.001

NA, not applicable; NR, not reached.

In order to compare the survival of ypII and ypIII rectal cancer patients, the survival curves were calculated together, which were stratified by response to nCRT (TRS 0–2) and no response. The overall survival of ypIII patients with response was not significantly different from ypII disease (**Figure 1C**, *P* = 0.56).

## Discussion

Based on previous studies, the tumor regression score after neoadjuvant chemoradiation was a significant independent prognostic factor for rectal cancer patients. Patients with no response to nCRT had the 5-year survival rate of 27% compared to 72% for patients with response (12). Similarly, in the CAO/ARO/AIO-94 trial, pCR patients had a 10-year disease-free survival of 89.5%, while those with poor regression had a corresponding incidence of 63% (13). The nCRT response had other predictive values in addition to predict the survival. In the EORTC 22921 trial, a subgroup analysis showed that ypT0–2 patients were more likely to benefit from adjuvant chemotherapy than ypT3–4 patients (8). Although the predictive value of the tumor regression score has been reported, a classification analysis of ypTNM stage has not been mentioned. Our current study analyzed the prognostic value of the tumor regression score classified by pathologic stage for the first time.

To investigate the impact of the tumor regression score on the classification stage, the patients were divided into two groups in each stage: response (TRS 0-2) and no response (TRS 3). The independent prognostic factor for ypII patients was histological grade. For patients at stage III who received nCRT, response to nCRT, well histological differentiation, negative surgical margin, and completion of adjuvant chemotherapy were all independently associated with improved survival. The differentiation and surgical margin but not the response to nCRT were consistent predictors of survival in both ypII and III patients. We found that ypIII patients with the response to nCRT had similar survival to that of ypII patients. However, it was difficult to distinguish the survival between response and no response among ypII patients.

It is known that surgical resection with negative margins in rectal cancer is critical because treatment modalities, such as chemotherapy and radiation, cannot compensate for a positive margin. The relationship between resection margin and local recurrence and survival has been reported by many studies. Our results substantiated the surgical margin as a prognostic factor of rectal cancer with nCRT. For histological tumor differentiation, well or moderate differentiation refer to cancer cells with a low invasive property. Poor differentiation is related to more aggressive cancer cells than the former. It is obvious that patients with poorer differentiated cancers suffer from worse oncologic outcomes in most cases.

It is necessary to investigate the reason for distinct outcomes of ypIII patients. According to the definition, ypIII tumors have cancer cells extending from the primary tumor location to the regional lymph nodes. Compared to ypII disease, ypIII tumors prefer to disseminate to other areas and are less likely to be solved by surgery alone. nCRT response could help us to discover the biology of tumors, which may act as an indicator of susceptibility to adjuvant chemotherapy. Both chemotherapy and neoadjuvant treatment could diminish recurrence by eradicating cancer cell that transferred to the lymphatic and blood vessels of patients with response to nCRT. Because of the resistance to cytotoxic agents, patients with no response had great possibility of recurrence. It was the inherent characteristics of the non-response ypIII patients that determined their prognosis. Whereas ypII tumors with disease located in the primary site and could be cured by surgery with a great possibility. This mostly clarified the mechanism that ypIII rectal cancer patients with response to nCRT had similar survival to ypII patients.

There were also many factors that could predict the degree of response. For ypII patients, there were statistical differences in gender and tumor size. Combined with clinical practice, we were unable to confirm whether the gender of patients was a prognostic factor of the response score, and the number of female patients was obviously less than males in our study. As for tumor size, it was evident that smaller tumors were more likely to show regression than larger tumors when treated by the same regimen. For ypIII patients, the predictive factors of TRS were clinical nodal status, stage, and the pathologic T stage. We were skeptical of this result because 65.9% of ypIII patients with T3 stage, and statistical bias could not be ignored. And the clinical stage showed a modest significance, which was unconvincing. Furthermore, we did not find a concurrent predictor of TRS in ypII and III diseases. Previous investigations have reported that TRS was associated with the interval between operation and nCRT completion, and patients with longer intervals were more likely to have lower tumor regression scores (14, 15). Most of the patients in our study underwent surgeries with interval about 8 weeks after the last time of chemoradiation. There was no difference of the interval between surgery and the last preoperative treatment of the patients, so we did not take it into consideration.

Regardless of the prognostic value of the TRS, its clinical implications are also important. In view of the poor response of some rectal cancer patients, they may not benefit from adjuvant cytotoxic therapy. However, with no alternative options, they could probably receive more intensive adjuvant therapies or participate in novel therapeutic trials. The panel of National Comprehensive Cancer Network (NCCN) believes that patients with tumor downstaging and complete response after nCRT should be strongly considered for adjuvant chemotherapy (8, 16). In addition to the choice of adjuvant therapy, the fact that long-term outcomes of the non-responders vary from patients with a response suggests that more rigorous surveillance is necessary for this population.

There were also some limitations of our study. Because this was a retrospective study, there was potential bias introduced by the loss of follow-up as well as from the variable collection of data. As part of the cancer database, our data were collected prospectively, which might help reduce the data bias to a certain extent. In addition, adjuvant chemotherapy regimens were not administrated based on the single treatment protocol. Some patients received CapeOX, whereas others received FOLFOX. The total pCR rate in our study was 12.1%, which was lower than the reported rates of 16-24% (16). This might be attributed to the dissimilarity of regimens and the generally late stage of rectal cancer patients in China. And this study reflects the outcomes of a specific population in China, and extending the results to other populations should be prudent.

## Conclusion

ypIII rectal cancer patients with poor response to nCRT are at high risk of worse oncological outcomes. More intensive adjuvant chemotherapy and surveillance may be performed in this population, and more effective approaches should be studied.

## Data Availability Statement

The raw data supporting the conclusions of this article will be made available by the authors, without undue reservation.

## Author Contributions

YY and HX participated in the design of the study, performed the data management, statistical analysis and interpretation and were co-primary investigators. GC and YP were major contributors to the design of this study and revised the manuscript. All authors contributed to the article and approved the submitted version.

## Conflict of Interest

The authors declare that the research was conducted in the absence of any commercial or financial relationships that could be construed as a potential conflict of interest.

## Publisher’s Note

All claims expressed in this article are solely those of the authors and do not necessarily represent those of their affiliated organizations, or those of the publisher, the editors and the reviewers. Any product that may be evaluated in this article, or claim that may be made by its manufacturer, is not guaranteed or endorsed by the publisher.
